# Developing sustainable system based on transformers algorithms to predict the Dubas insects diseases in palm leaves

**DOI:** 10.3389/fpls.2025.1612800

**Published:** 2025-09-04

**Authors:** Theyazn H. H. Aldhyani, Hasan Alkahtani

**Affiliations:** ^1^ Applied College, King Faisal University, Al-Ahsa, Saudi Arabia; ^2^ College of Computer Science and Information Technology, King Faisal University, Al-Ahsa, Saudi Arabia

**Keywords:** palm, diseases, transformers, deep learning, sustainable, insect

## Abstract

**Introduction:**

Agriculture has emerged as a crucial area of inquiry, presenting a significant challenge for numerous experts in the field of computer vision. Identifying and categorizing plant diseases at an early stage is essential for mitigating the spread of these diseases and preventing a decline in crop yields. The overall condition of palm trees, including their roots, stems, and leaves, plays a crucial role in palm production, necessitating careful observation to ensure maximum yield. A significant challenge in maintaining productive crops is the widespread presence of pests and diseases that affect palm plants. The impact of these diseases on growth and development can be significantly negative, resulting in reduced productivity. The productivity of palms is intricately linked to the state of their leaves, which are essential for the process of photosynthesis.

**Methods:**

This study utilized an extensive dataset comprising 1600 images, which included 800 images of healthy leaves and another 800 of Dubas images. Additionally, the primary aim was to develop EfficientNetV2B0, DenseNet12, and a transformer model known as the Vision Transformer (ViT) model for detecting diseases and pests affecting palm leaves, utilizing image analysis methods to enhance pest management strategies.

**Results:**

The proposed models demonstrated superior performance compared to numerous recent studies in the field, utilizing established metrics on both original and augmented datasets, achieving an impressive accuracy of 99.37% with the ViT model.

**Discussion:**

This study presents an innovative approach for identifying diseases in palm leaves. This will have a significant impact on the agricultural sector. The results were quite promising, justifying their implementation in palm companies to improve pest and disease management

## 1 Introduction

Round 70% of the world’s date fruit comes from Saudi Arabia, thanks to its more than thirty-one million palm trees (The Ministry of Environment and Water and Agriculture Saudi Arabia, 2020). In 2023, the more than 26,000 date farms located in the Madinah area of western Saudi Arabia produced about $253 million. Roots, trunks, leaves, and fruits of palm trees are all vulnerable to a host of infectious illnesses caused by bacteria and fungi (The Saudi Press Agency (SPA), 2024).

The inventory of palm trees is essential for assessing diversity and health; however, data regarding their numbers and distribution is limited, outdated, and inconsistent. Estimations of date palm trees in plantations rely on assessments that exclude non-agricultural areas and natural populations. Data on canary palm populations is restricted to areas of significant public interest, with estimates derived from date palm production rather than geospatial databases (Jaradat, 2015; Sharma et al., 2021; Zaid and de Wet, 2002).

Agriculture, and date palm trees in particular, are very vulnerable to diseases and climate change. Date palm trees suffer greatly from serious diseases, including Brittle Leaf disease, Brown Leaf Spot, Bayoud disease, Black Scorch, SDS, and Brown Leaf Spot, which drastically reduce fruit quality and productivity. This study examines the detection of the SDS disease, which disseminates in a hazardous manner, complicating control efforts and resulting in substantial losses in fruit output. This disease poses a significant threat to Date palm farming worldwide and hinders fresh planting efforts (Khamparia et al., 2020).

Palm leaves are integral to numerous ecosystems, economies, and civilizations. They play a crucial role in various aspects, supporting individuals in earning a livelihood and staying on their chosen course in life. Nevertheless, the Dubas bug presents a significant risk to date palm trees and their leaves, especially in areas where date palm farming is prevalent. Identifying and classifying plant diseases is crucial for precision agriculture; however, farmers face challenges in diagnosing these diseases and assessing the extent of infestation. The combination of machine vision and deep learning has revolutionized the automated detection and assessment of pests and diseases in agriculture. Palm leaf diseases pose a considerable challenge to the health and productivity of trees (Resh and Cardé, 2009; Hessane et al., 2023).

Adult, nymph, and egg are the three phases that make up a dubas bug’s life cycle. With its two sets of wings, this insect is hemimetabolous. It undergoes a mating cycle in the spring (February–May) and another in the fall (August–November) of each year. Date palm females use the third and fifth leaf fronds, respectively, to deposit their eggs in the spring and autumn.

The life cycle begins with oviposition, followed by hatching into nymphs and undergoing five molts until the adult form is attained. Adults have a yellowish-brown to greenish coloration, characterized by two black patches on their heads. They generate honeydew and necrotic regions in plant tissues due to their oviposition behavior. Nonetheless, it remains uncertain whether these necrotic lesions result from fungal infections (Elwan and Al-Tamimi, 1999; Thacker et al., 2003; Ba-Angood et al., 2009; Resh and Cardé, 2009; Payandeh et al., 2010).

Computer-based technologies are rapidly revolutionizing agriculture, reducing human labor, and enabling impartial decision-making. Image processing methods are utilized in various computer vision applications for disease diagnosis, identification, and segmentation tasks. This technological development is revolutionizing agriculture, improving efficiency and efficacy. Using a modified MobileNetV2 neural network, the authors (Kong et al., 2022) enhanced the accuracy of cassava leaf disease classification by employing data augmentation methods on lower-quality test images.

With high-quality photographs, they achieved a 97% recognition accuracy; however, this accuracy was significantly reduced with low-quality images. Using a range of classifiers at the image level, including Fine KNN, Cubic SVM, and tree ensemble, the authors in (Li et al., 2023) classified guava plant illnesses with an overall classification accuracy of 99%. Plant disease classification is achieved by a hybrid wrapper model that combines CNN classifiers with FPA-SVM (Bayomi-Alli et al., 2021). This methodology yielded a classifier with an accuracy of 99% through feature selection using FPA and SVM in a wrapper approach. The authors (Almadhor et al., 2021) proposed a deep learning (DL) model for disease detection on cucumber and potato leaves, utilizing an optimization approach. A 99% success rate was achieved by optimizing the DFs obtained from the global pooling layer using an upgraded Cuckoo search strategy. For disease categorization in plant leaves, Yağ and Altan (2022) presented the EfficientNet DL architecture. They employed a transfer learning approach to train their model and other deep learning models, and both performed well. To classify citrus diseases, Zia Ur Rehman et al. (2022) developed a new DL model. The accuracy rate was 95% because the Whale Optimisation Algorithm (WOA) was used to retrain two pre-trained models, DenseNet 201 and MobileNetv2, so that they could produce feature vectors. A DL model for guava disease identification has achieved a 97% accuracy rate by utilizing enhanced data supplemented with color-histogram equalization and unsharp masking techniques (Atila et al., 2021). The primary contributions of this research are as follows.

Make a substantial contribution to the categorization of palm leaf diseases by using cutting-edge deep learning architecture. This paper presents a method for the automated detection and enumeration of palm leaves, disease identification, and assessment of palm health from high-resolution images using deep learning and Vision Transformer models. The training dataset included more than 1600 images of individual dubas and healthy classifications. We assessed the model via training assessment and by comparing prediction outcomes with visual and ground inspections. The model was also evaluated using images captured at varying elevations. It can achieve elevated accuracy with less labeled data by using pre-trained models. This method enhances classification efficacy while reducing training duration and computational expenses. Additionally, it streamlines the process of disease identification, providing a scalable and rapid option for early detection. This is essential for agricultural disease management.

## 2 Related works

Several new approaches to identifying and categorizing plant diseases have recently been developed. The methods were tested on a variety of datasets, each with its distinct features. Representing plant photos using practical and discriminative characteristics is essential for creating a system to identify plant diseases. There are two primary schools of thought regarding feature extraction methods: those that rely on manually created features and those that utilize deep learning techniques (Shah et al., 2016; Sethy et al., 2020; Iqbal et al., 2023; Tholkapiyan et al., 2023).

Using CNN with SVM, Kamal et al. (2018). We were able to differentiate between Chimara (the most prevalent date palm spot leaf disease) and Anthracnose (the least frequent), achieving accuracy rates of 97% and 95%, respectively. To attain a classification accuracy of 99.67% with an artificial neural network (ANN) classifier, Hamdani et al. (2021) suggested an approach that makes use of a color histogram feature and a dataset consisting of 300 laboratory photos. With an overall accuracy of above 96%, Liu et al. (2021) used a DL-based Faster RCNN to identify and quantify oil palm plants in UAV pictures. Abu-zanona et al. (2022) achieved the best accuracy for the Kaggle dataset, classifying four types of sick palm trees with 97% accuracy using VGG 16 and MobileNet.

Septiarini et al. (2021) presented a method for detecting diseases in oil palm leaves. Otsu thresholding was employed in the Lab color space to identify Regions of Interest (ROIs), followed by preprocessing and classification using k-nearest neighbors (KNN) (Eunice et al., 2022). The identification of leaf diseases in tomato plants was conducted by Sunil S. Harakannanavar et al (Harakannanavar et al., 2022). This approach combines multiple techniques, such as SVM, KNN, and CNN, for the detection of palm diseases. Recent studies have developed a CNN for disease classification in palm trees (Abu-zanona et al., 2022).

Authors (Nusrat et al., 2020) were able to detect and categorize wheat illness leaves with a 98% success rate using SVM and GoogLeNet, two ML models. A prior study by (Too et al., 2019) compared several DL models for plant disease detection. This study utilized 121-layer customized models, including VGG-16, Inception V4, ResNet, and DenseNets, to classify plant species. This study used the model to detect and classify healthy leaves, as well as four prevalent diseases that may affect palm trees: bacterial leaf blight, brown spots, leaf smut, and white scale. When tested against VGG-16 and MobileNet, two prominent CNN models, the suggested model outperformed them both with an accuracy rate exceeding 99%. In a separate study, Hamdani et al. (2021) examined methods for identifying and categorizing diseases that affect palm trees. The authors of this study achieved a 99% success rate in classifying palm diseases using an artificial neural network (ANN) classifier and principal component analysis (PCA) to extract color features.

Masazhar and Kamal (2017) employed an automated system to detect and categorize disease indicators in palm oil leaves. Two palm oil illnesses were successfully detected using k-means clustering, an SVM classifier, and leaf symptomatology. Thirteen novels were produced from k-means clustering for disease classification. Ashqar and Abu-Naser (2018) utilized a CNN model for identifying tomato leaf diseases, demonstrating improved performance with full-color images compared to grayscale images. Shruthi U et al. highlighted the efficacy of convolutional neural networks in identifying specific agricultural diseases through machine learning methodologies (Shruthi et al., 2019).

## 3 Materials and methods

### 3.1 Farmwork of the proposed system

**Figure 1:**
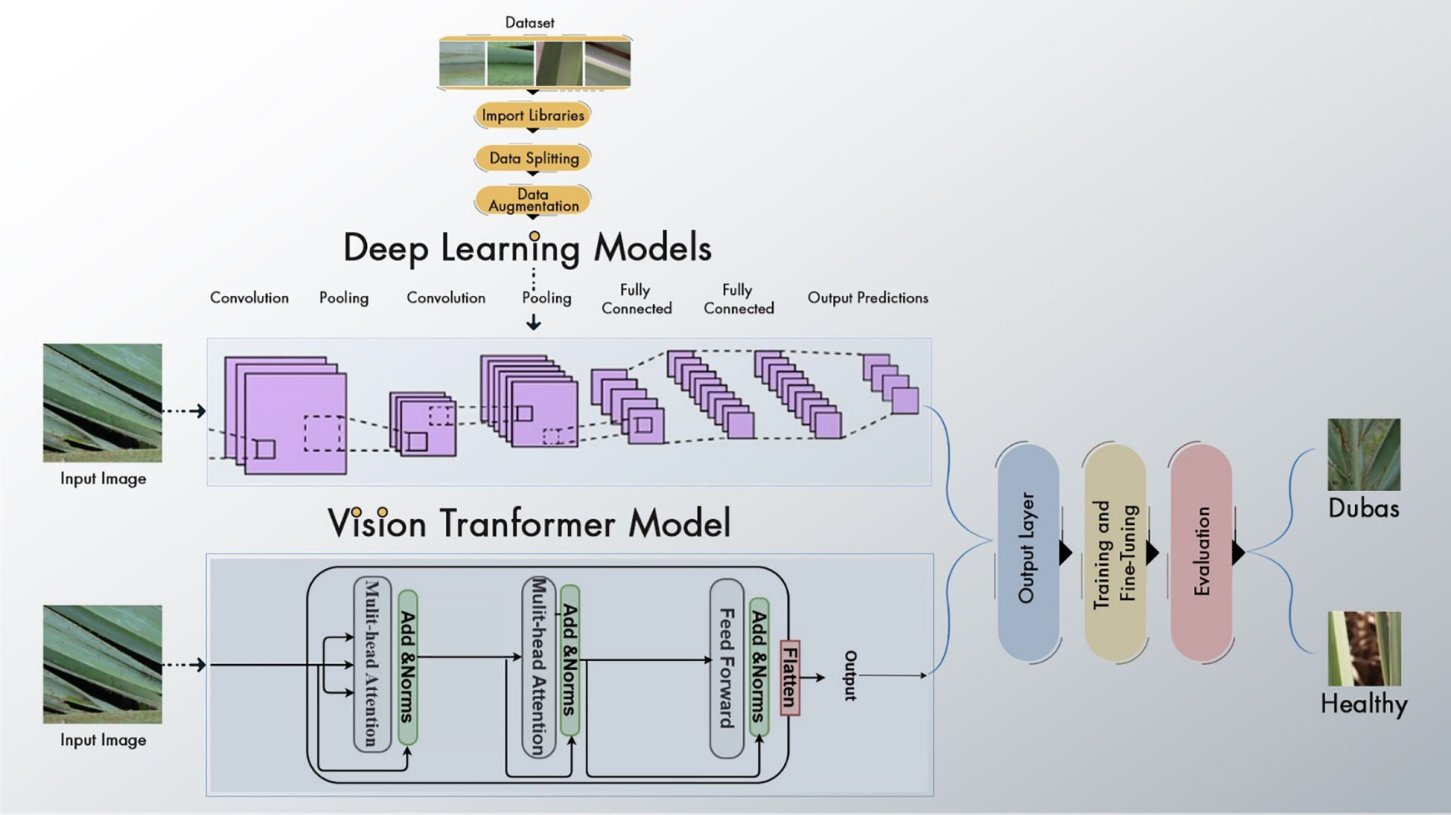
Farmwork of the palm system.

The suggested paradigm for disease detection in palm leaves is shown in **Figure 1**. The proposed framework covers the following crucial steps. The data is first enhanced to improve the training process by increasing the sample count. Then, we choose and improve EfficientNetV2B0, DenseNet12, and Vision Transformer. ViT features are obtained from the global pooling layer, and deep learning further trains the models. We conclude by drawing parallels to popular and up-to-date DL and transformer kinds. Training Vision Transform models using the Palm-leaves dataset is the focus of this research.

### 3.2 Dataset

**Figure 2:**
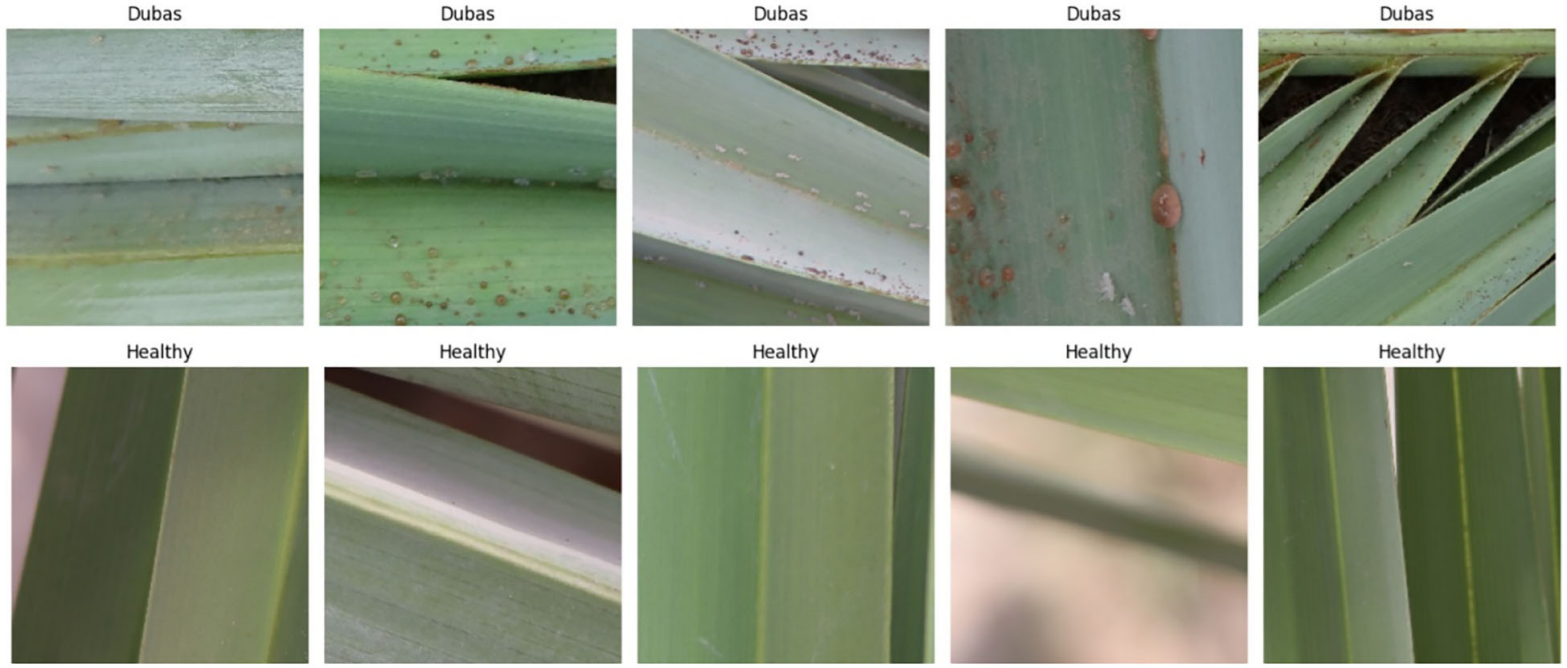
Sample from palm data.

**Figure 3:**
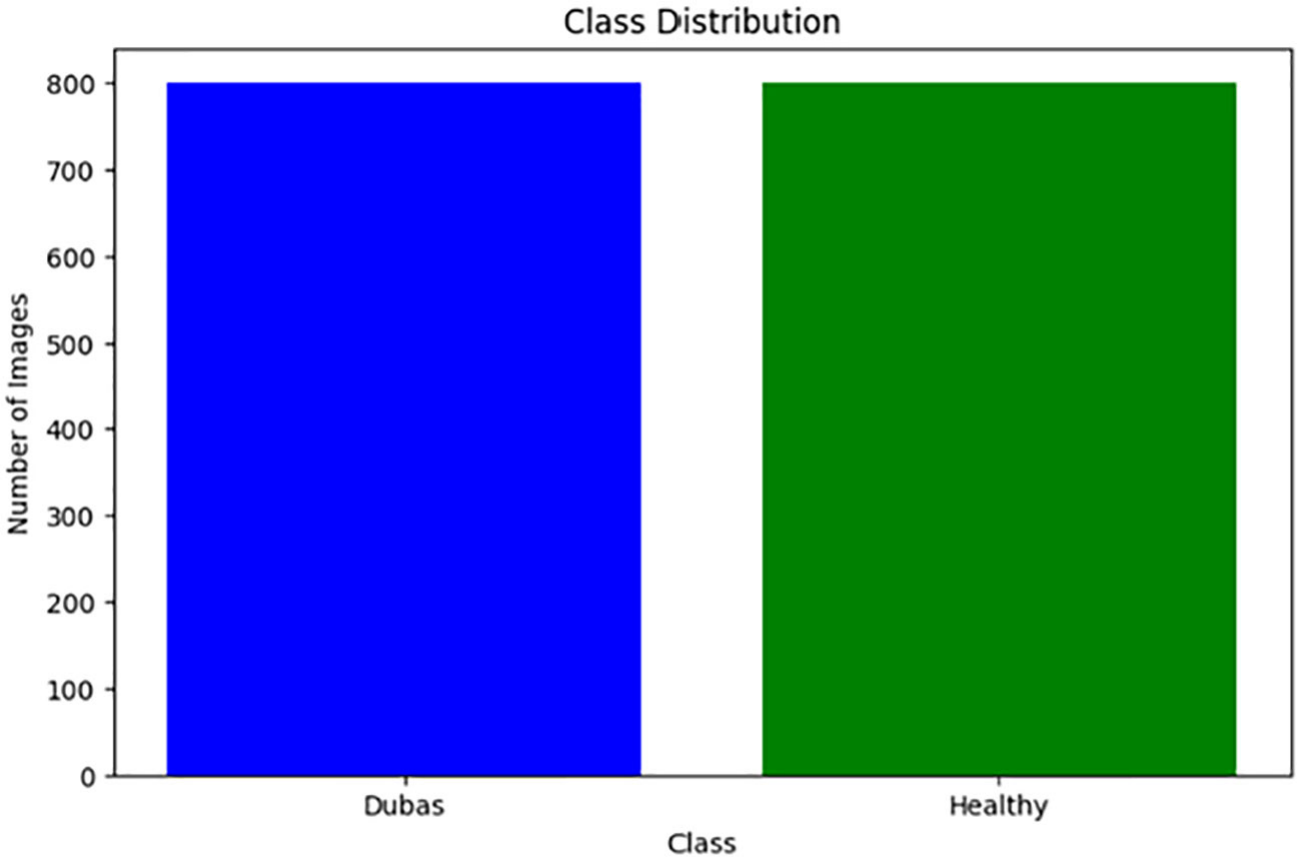
Class of palm data.

We obtained the dataset from the Karbala Governorate in Iraq via Kaggle, collecting leaves to varying degrees. In the research, we used palm leaves with images of Dubas and health. The image resolutions are 6000 × 4000 × 3 pixels for the Canon 77D camera and 8000 × 6000 × 3 pixels for the DJI Camera 800 images of Dubas and 800 images of the health class. A snapshot of the palm leaves is presented in **Figure 2**. **Figure 3** presents the class values of the palm leaves dataset.

### 3.2 Preprocessing steps

During the preprocessing phase of EfficientNetV2B0, DenseNet121, and ViT, several critical processing steps were implemented to ensure high-quality input data for model training. Initially, all images were downsized to a consistent dimension suitable for each model’s architecture: 224×224×3 for EfficientNetV2B0 and DenseNet121, and 384×384×3 for ViT. Subsequently, the pixel values of palm images were standardized using the mean and standard deviation to enhance model convergence. These models used data augmentation methods, including rotation, flipping, zooming, and contrast modification, to improve model generalization and flexibility. Furthermore, the ViT model uses the tokenization of image patches before processing, whereas CNN-based models employ feature scaling to ensure consistency. These preprocessing measures enhance model efficiency, accuracy, and generalizability.

### 3.3 Proposed models

#### 3.3.1 EfficientNet-B0 model

**Figure 4:**
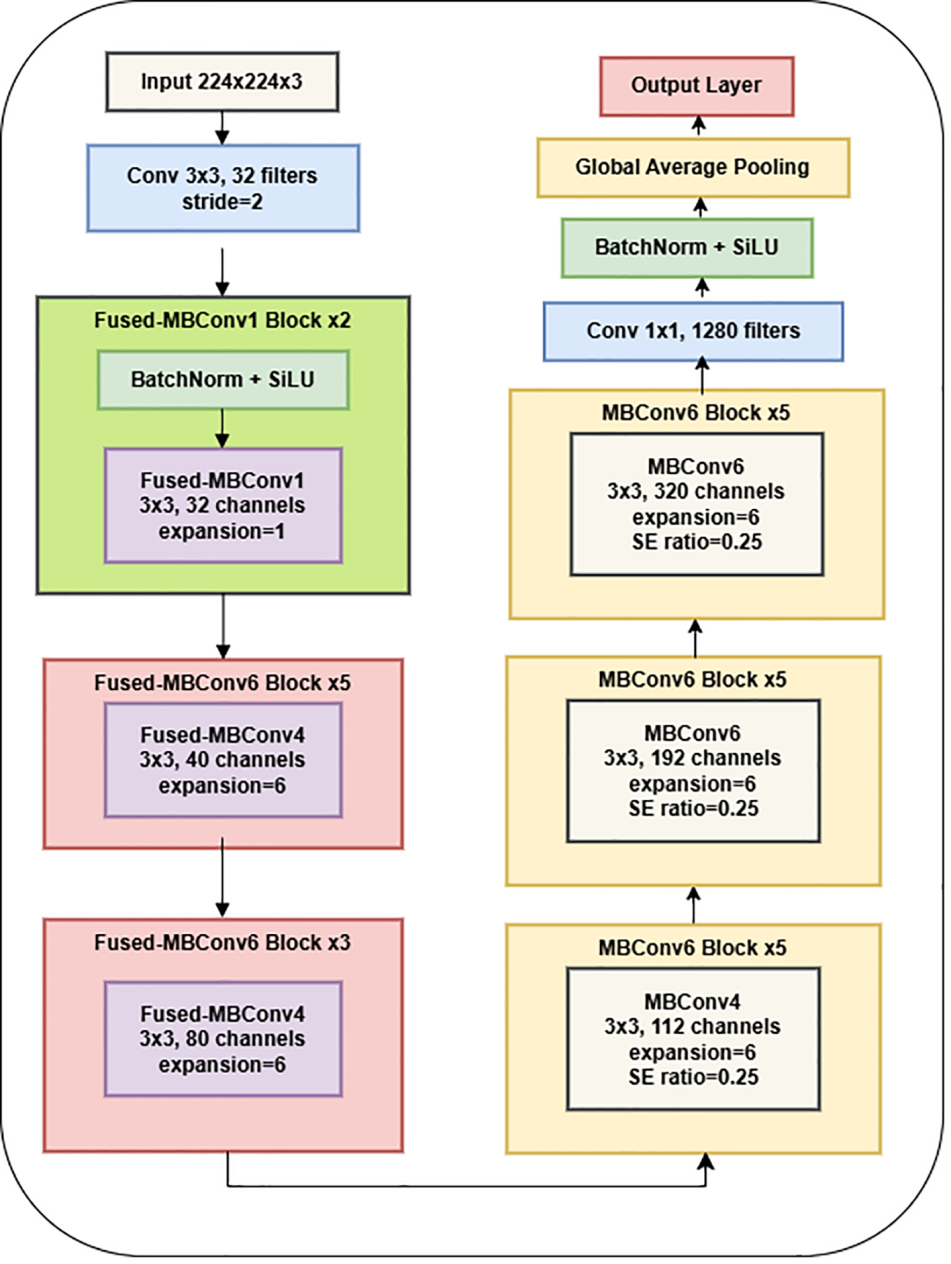
EfficientNetV2B0 model.

The EfficientNet-B0 architecture is a well-established CNN model that can serve as an encoder in tasks involving semantic segmentation. EfficientNet-B0 served as the backbone network in the proposed research design for feature extraction from the input image through downsampling. EfficientNet-B0 is a CNN architecture comprising several blocks, each incorporating convolutional layers, activation functions, and pooling operations. This architecture is a convolutional neural network commonly employed for image classification tasks. The output of EfficientNet-B0 is frequently used as input for a decoder network in semantic segmentation. The application of EfficientNet-B0 as an encoder for semantic segmentation has demonstrated remarkable accuracy and efficiency in various contexts, particularly in medical image segmentation (Hamdani et al., 2021; Liu et al., 2021). **Figure 4** presents the plots generated by the encoder model.

The network is optimized for classification (healthy vs. Dubas) through the incorporation of a global average pooling layer and a dense layer utilizing a sigmoid activation function. The model was trained on augmented image data that had horizontal flips. An adaptive learning rate and early stopping were used to avoid overfitting. Using depthwise separable convolutions, batch normalization, and activation layers, the EfficientNetV2B0 architecture has 236 layers that are optimized for effective feature extraction. A transfer learning framework utilizes the pre-trained base, omitting the upper classification layers. Along with the base model, two extra layers are added: a Global Average Pooling layer that combines spatial data from feature maps and a Dense layer that uses a sigmoid activation function for binary classification. The model comprises 238 layers and combines EfficientNetV2B0’s powerful feature-extraction capabilities with a simplified custom classification head designed for binary classification tasks. **Table 1** displays the parameters of the EfficientNet-B0 model.

**Table 1:** Parameter EfficientNet-B0 model.

#Name	#Values
Layers	238
Image	224x224x3
Optimize	Adam
Learning_rate	0.001
Batch_Size	16
Epochs	20

#### 3.3.2 DenseNet121 model

**Figure 5:**
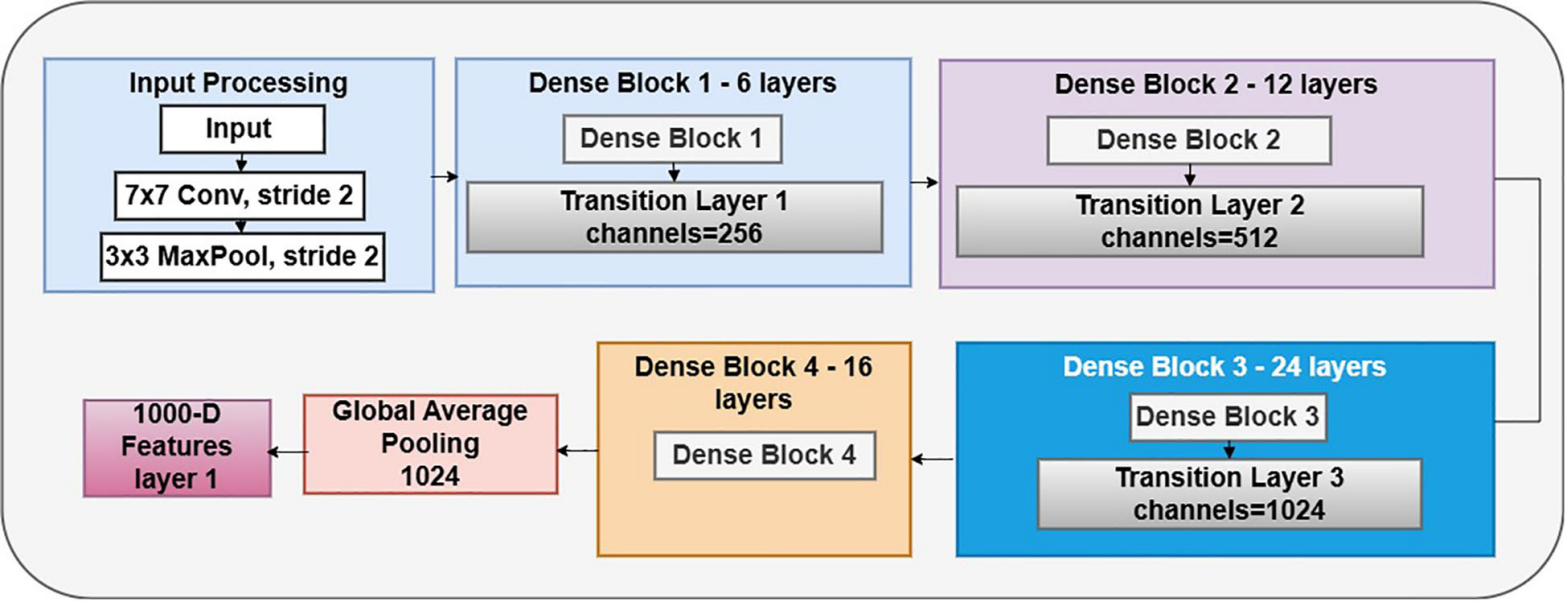
DenseNet121 model.

DenseNet121 is a CNN architecture proposed by Huang et al. It belongs to the DenseNet family, recognized for its dense network architecture and remarkable efficacy in several computer vision applications, including image categorization. The design of DenseNet-121, as shown in **Figure 5**, is centered on the principle of dense connections. Unlike standard CNN designs, where layers are sequentially linked, DenseNet utilizes skip connections that link each layer to every other layer in a feed-forward way. This intricate connection structure facilitates direct feature reuse and promotes information flow throughout the network, leading to improved gradient propagation, enhanced feature extraction, and overall model efficacy. The core feature extractor is DenseNet-121, a pre-trained convolutional neural network developed for image classification tasks. Across all 121 levels, this design effectively utilizes gradient movement and feature reuse due to its rich connections. Class weights, a binary cross-entropy loss function, and the Adam optimizer, which has a learning rate of 0.001, are used by the model to handle data imbalances. Metrics, including confusion matrices, classification reports, and accuracy, are used for evaluation. Essential parameters are a batch size of 16, a target image dimension of (224, 224), and 20 training epochs.

#### 3.3.3 Vision transformer model

The Vision Transformer (ViT) is an innovative neural network design that transforms the processing and comprehension of images. The Vision Transformer (ViT) concept was presented in 2021 in a conference research paper entitled “An Image is Worth 16*16 Words.” Transformers for Image Recognition at Scale, or ViT, presents an innovative approach to image analysis by segmenting images into smaller patches and using self-attention processes. This enables the model to discern both local and global links among images, resulting in remarkable performance across many computer vision tasks. Whereas CNNs immediately analyze raw pixel values, ViT segments the input image into patches and converts them into tokens. ViT utilizes self-attention processes to analyze connections among all patches. The Vision Transformer (ViT) inherently captures global context through self-attention, enabling the recognition of relationships among distant patches. Convolutional Neural Networks use pooling layers to extract coarse global information.

**Figure 6:**
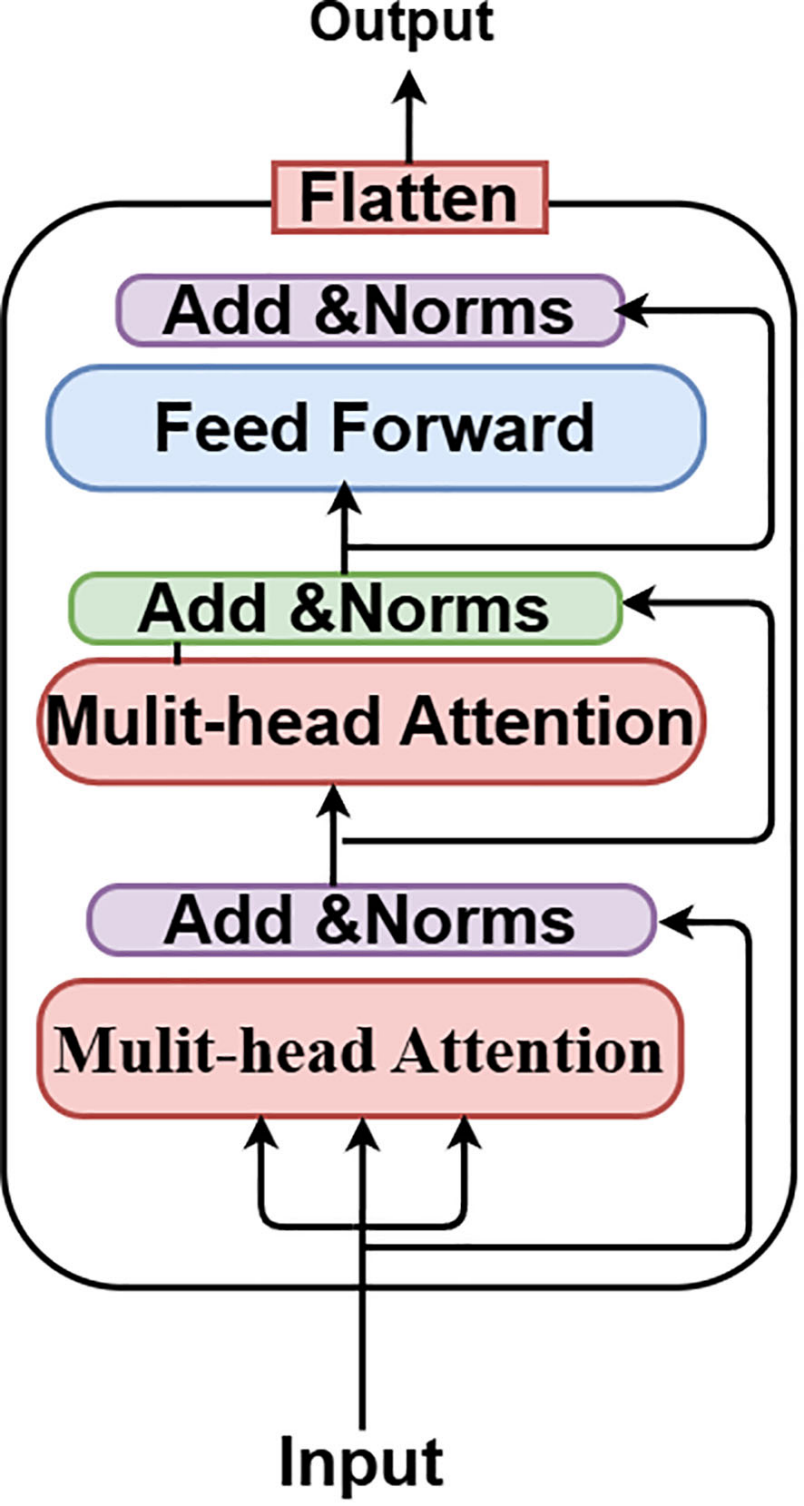
ViT model.

It can be fine-tuned for individual tasks after pre-training on large datasets. By segmenting 224x224 input images into patches and modeling their interrelationships using self-attention mechanisms, the ViT model can perform analysis. The B16 architecture is used by this ViT model, as seen in **Figure 6**. Feature extraction is made easier with its transformer block, which incorporates feedforward neural networks and many attention layers trained on massive datasets. An integrated custom classification head, along with immobilized pre-trained weights and layers, ensures the model’s obtained representations remain intact. This head comprises an activation layer with a regularization rate of 0.5, a dense layer with 128 neurons activated by ReLU, and a final dense layer with sigmoid activation for binary classification. Class weights are used to reduce class imbalance, and adjustments to data augmentation methods improve the model’s generalizability. After 20 iterations of training using the Adam optimizer at a learning rate of 0.001, the model is complete. The ViT values are displayed in **Table 2**.

**Table 2:** ViT parameters.

#Name	#Values
Patch_size:	16,
Transformer _encoder:	12
Attention_heads	12
Hidden_size	768
Optimizer:	Adam
Epochs:	20
Learning_Rate:	0.001
Image_Size	244X244
Dropout_Rate:	0.5

## 4 Experiments and results discussion

The experiments were evaluated using a GPU P100 Kaggle system as the baseline. Operating Windows 11, the machine features 16 GB of RAM and a 9th-generation Core i7 CPU. Aiming to enable deep learning applications with reduced memory usage and improved execution speed, the software implementation included libraries such as Anaconda, Keras, OpenCV, NumPy, and CuDNN. For every experiment carried out, this work assessed the training and testing accuracy. Every model has calculated losses throughout the testing and training periods. Training the models on the Palm tree dataset helped to improve the learning speed of the transformer and transfer learning models. EfficientNetV2B0, DenseNet121, and ViT models were used for this work.

Each of the two dataset classes corresponded to a different disease. Since the Palm dataset’s color images worked well with the DL and ViT models, we used them in our experiments. The images were downscaled to a uniform pixel format since different pre-trained network models require varied input sizes. Input dimensions of 224 × 224 × 3 (height, width, and channel depth) are used by EfficientNet V2B0, DenseNet 121, and ViT. Data augmentation after preprocessing is a regularization strategy that is used to reduce the impact of overfitting. This method of model augmentation makes the model more robust, which in turn enhances its ability to categorize images of real plant diseases while reducing the likelihood of overfitting and model loss.

### 4.1 Results

#### 4.1.1 Result of EfficientNetV2B0 model

The findings of the EfficientNetV2B0 model, presented in **Table 3**, demonstrate robust performance across all assessed criteria, indicating a highly efficient classification system. The model demonstrates balanced and consistent performance in accurately detecting instances of both the “Dubas” and “Healthy” classes, with accuracy, recall, and F1-score all at 98%. The total accuracy of 98% further substantiates the model’s reliability. The weighted average of accuracy, recall, and F1-score, again at 98%, indicates that the model generalizes well across the dataset, maintaining excellent performance without significant bias towards any one class. The findings underscore the efficacy of the EfficientNetV2B0 model for this classification job, demonstrating exceptional predicted accuracy and dependability.

**Table 3:** Performance of EfficientNetV2B0 model.

Class name	Precision (%)	Recall (%)	F1-score (%)	Support samples of/validation phase
Dubas	96	100	98	80
Healthy	100	96	98	80
Accuracy		98		
Weighted_Avg_ palm system	98	98	98	160

**Figure 7:**
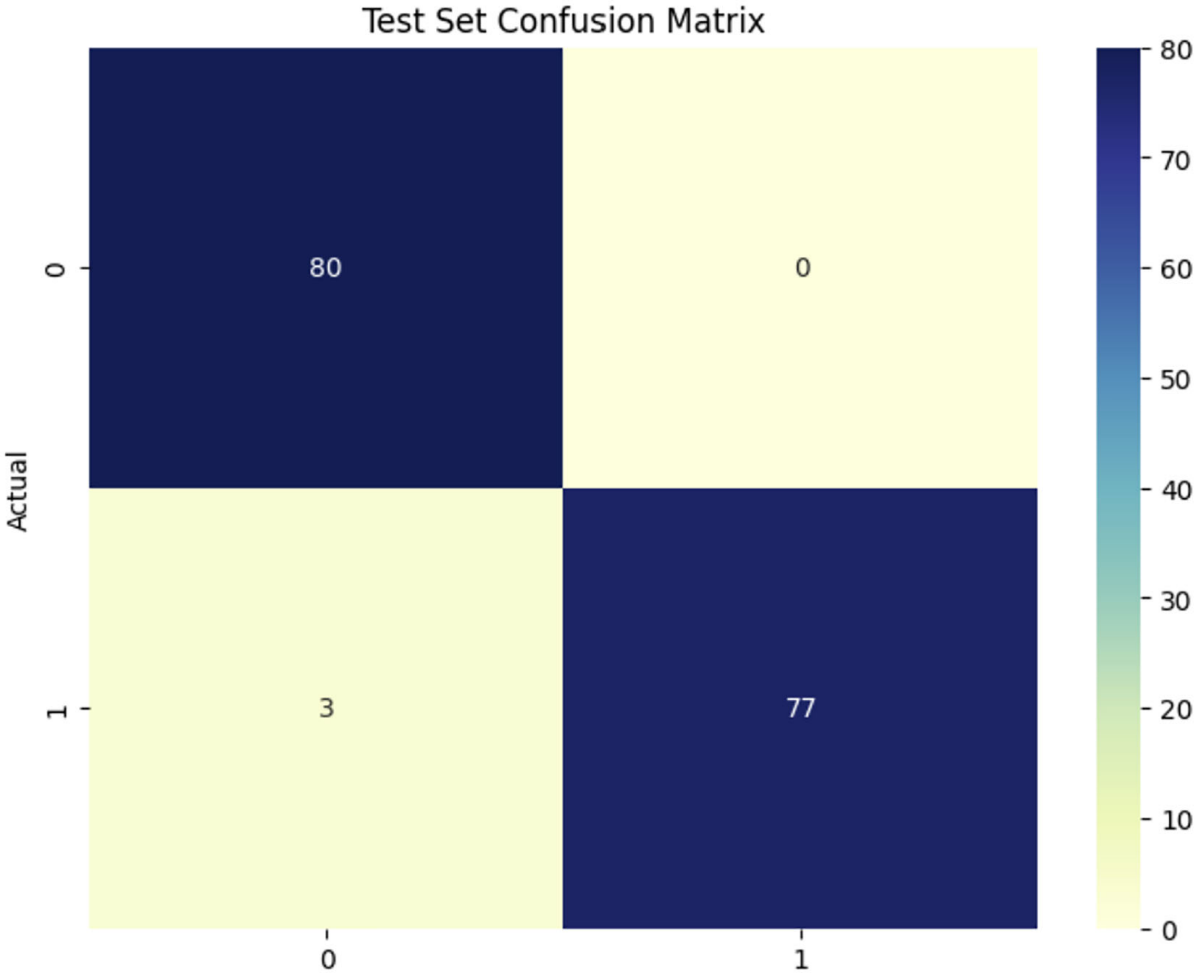
confusion matrix EfficientNetV2B0 model.

**Figure 7** illustrates the confusion matrix for the EfficientNetV2B0 model used in a classification job differentiating between healthy and Dubas samples, demonstrating outstanding performance. The model analyzed 160 test samples (80 each class), accurately identifying 80 healthy and 77 Dubas samples, resulting in 3 misclassifications from each class, yielding 3 false positives and 0 false negatives. This yields an exceptional accuracy of 98.12%, demonstrating the model’s robust discriminative capability. Nonetheless, the almost flawless classification requires further validation on an independent dataset to verify its robustness and alleviate concerns over possible overfitting.

#### 4.1.2 Result of DenseNet121 model

**Table 4** illustrates that the retrained DenseNet121 model exhibits strong performance in both classes, as evidenced by the elevated accuracy, recall, and F1 Scores. For the Dubas class, the model achieves a precision of 99%, indicating that 97% of the examples predicted as Dubas are accurate, and a recall of 97%, demonstrating that the model recognizes 97% of all genuine Dubas occurrences. In the Healthy class, the accuracy and recall are 97% and 99%, respectively, indicating the model’s proficiency in reliably classifying healthy samples. The F1-scores for both groups are 98%, indicating a balanced equilibrium between accuracy and recall. The model’s overall accuracy of 98% highlights its success, accompanied by a weighted average precision, recall, and F1-score of 98%, demonstrating consistent performance across the dataset. The findings indicate that the DenseNet121 model has shown high performance.

**Table 4:** Result of DenseNet121 model.

Class name	Precision (%)	Recall (%)	F1-score (%)
Dubas	99	97	98
Healthy	98	99	98
Accuracy		98	
Weighted_Avg	98	98	98

**Figure 8:**
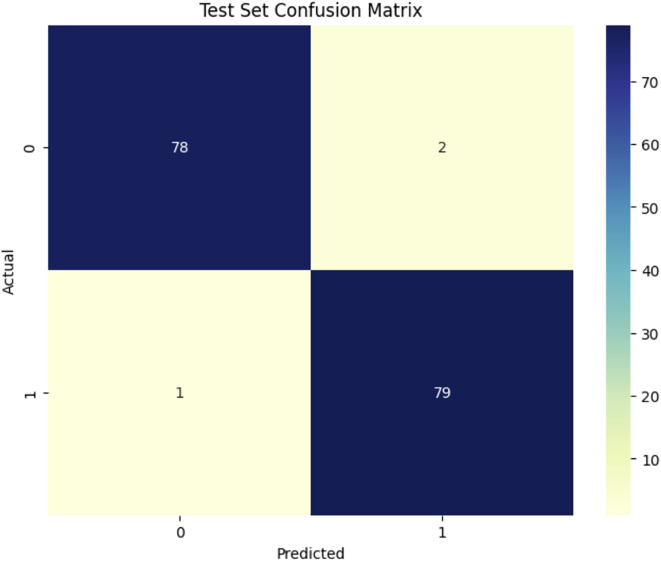
Confusion matrix DenseNet121model.

**Figure 8** shows the confusion matrix for the DenseNet121 model in classifying healthy (1) and Dubas (0) data, demonstrating outstanding performance. Out of 160 test samples (80 in each class), the model correctly names 78 as Dubas and 79 as healthy. This led to three mistakes: two false positives (healthy samples were mistakenly labeled as Dubas) and one false negative (Dubas samples were labeled as Healthy). This yields an accuracy of 98.12%, demonstrating the efficacy of DenseNet121 in feature extraction and classification. The modestly reduced misclassification rate indicates a modest improvement in generalization.

#### 4.1.3 Result of ViT model

**Table 5** presents the performance parameters of the ViT model in distinguishing between Dubas (0) and Healthy (1) samples, highlighting its robust predictive potential. The precision for Dubas is 100%, indicating that almost all samples categorized as Dubas are accurate. Meanwhile, the recall is 97%, demonstrating that 96% of genuine Dubas samples were accurately recognized. Correspondingly, for the Healthy class, the model achieves 98% accuracy and 100% recall, ensuring that most Healthy samples are accurately identified. The F1-score, which equilibrates accuracy and recall, is 99% for both classes, underscoring the model’s overall dependability. The overall accuracy of 99% and a weighted average F1-score of 99% indicate that ViT exhibits uniform classification performance across both categories. The findings validate the model’s efficacy in differentiating between Dubas and Healthy instances, exhibiting only negligible misclassifications, hence underscoring its robustness and generalizability in medical image classification.

**Table 5:** ViT model performance.

Class name	Precision (%)	Recall (%)	F1-Score (%)
Dubas	100	97	99
Healthy	98	100	99
Accuracy		98	
Weighted_Avg_plam system	99	99	99

**Figure 9:**
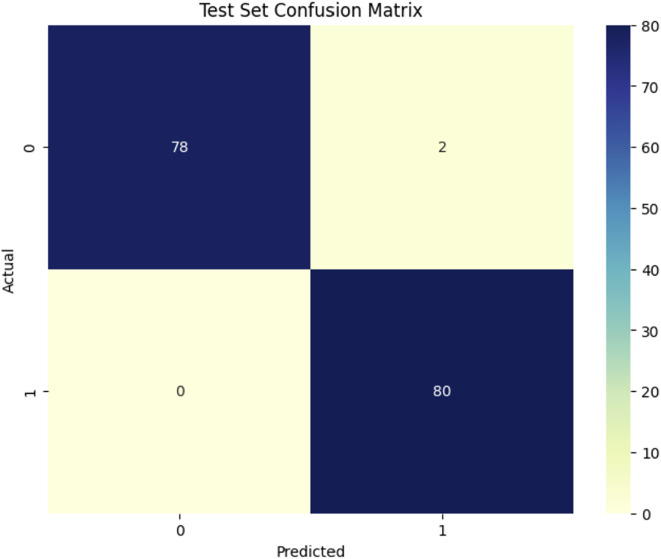
ViT model.

**Figure 9** illustrates the confusion matrix for the ViT model in identifying Dubas (0) and Healthy (1) samples, indicating its excellent accuracy. The model accurately categorized 78 Dubas samples, misclassifying only 2 as Healthy, resulting in a high recall for the Dubas category. It accurately recognized all 80 Healthy samples without any false negatives, indicating that every true Healthy instance was found. This signifies that the model exhibits perfect recall for the Healthy class. However, its small misclassification in the Dubas group implies a little compromise in specificity. The ViT model demonstrates robust prediction performance with few mistakes, making it extremely dependable for differentiating between Dubas and Healthy instances.

### 4.2 Results performance

This segment of the research used cutting-edge deep learning and Vision Transformer models for the identification of palm leaf diseases. Previously trained on the ImageNet dataset, the publicly available Palm Leaves dataset was utilized to augment pre-trained deep learning (DL) and Vision Transformer (ViT) networks. Every model in our study was standardized using two output classes, a dropout rate of 0.5, and a learning rate of 0.001.

**Figure 10:**
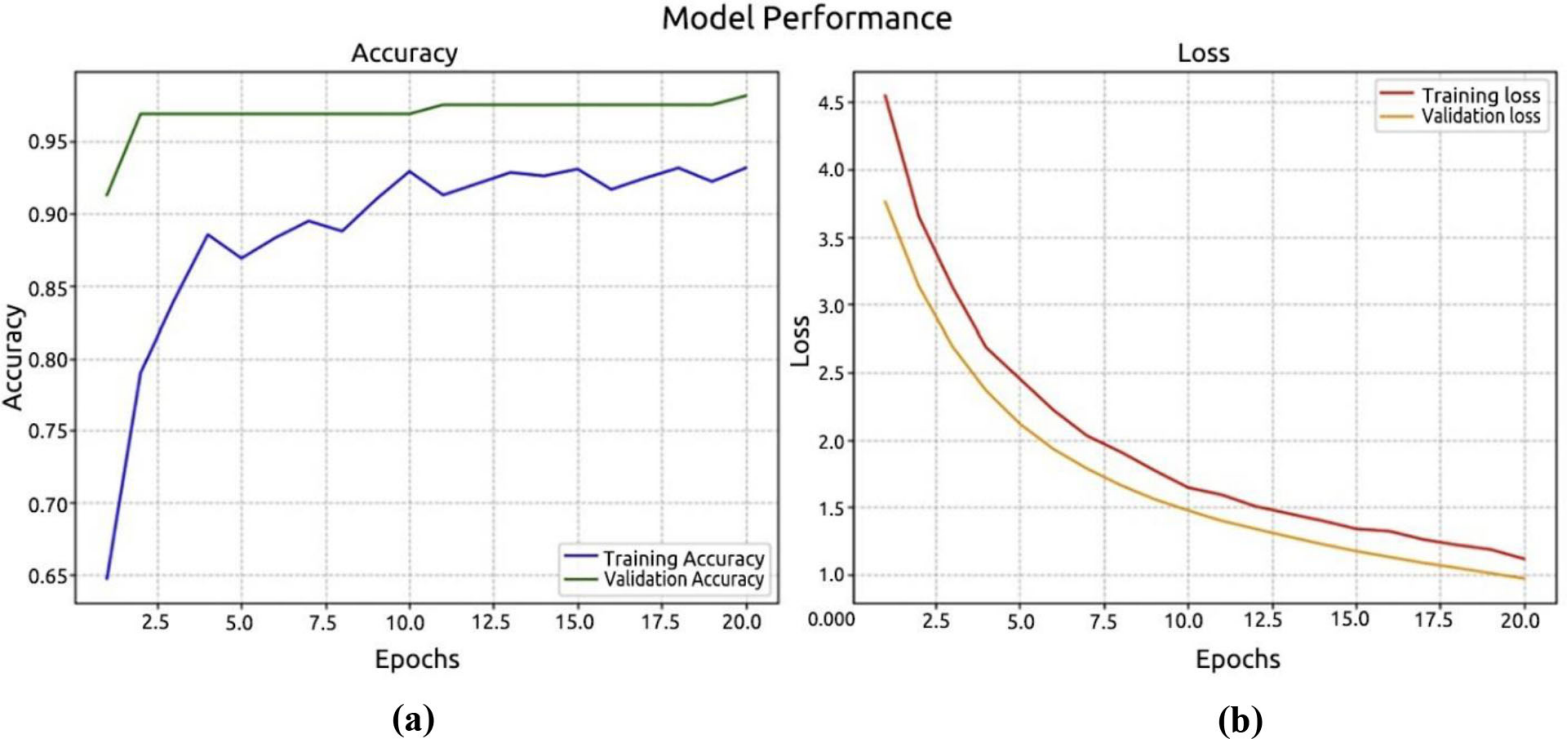
Performance of the EfficientNetV2B model. **(a)** accuracy; **(b)** loss.

The dataset consisted of training, testing, and validation samples. Of the palm leaf samples, 80% were set aside for pre-training EfficientNetV2B0 models. Every model run for 10 epochs and showed that our model started to converge with high accuracy after this length. The first experiment demonstrates the performance of the EfficientNetV2B0 model, as illustrated in **Figure 10a**. **Figure 10b** displays the log loss of the EfficientNetV2B0 model. The EfficientNetV2B0 model reached a testing accuracy of 98.12%.

**Figure 11:**
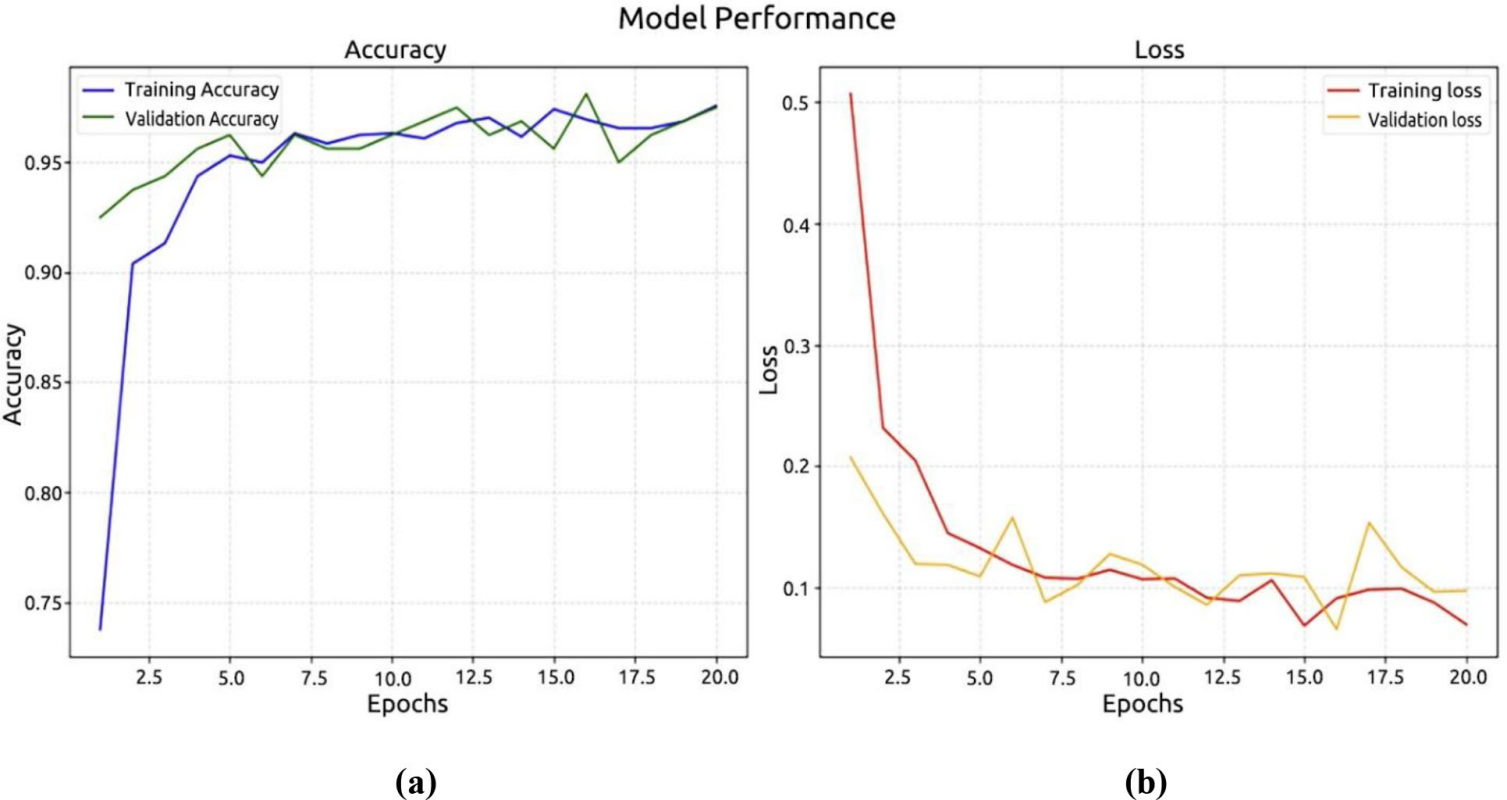
Performance DenseNet121 model. **(a)** accuracy; **(b)** loss.

In the second experiment, we used the Palm dataset to test DenseNet-121. Based on **Figure 11a**, the model achieved a recognition accuracy of around 97.50% in the first 10 epochs, and then it increased to a high accuracy of 92.50%. At 0.20%, the recorded loss model and at 0.0974%, the validation model are shown in **Figure 11b**.

**Figure 12:**
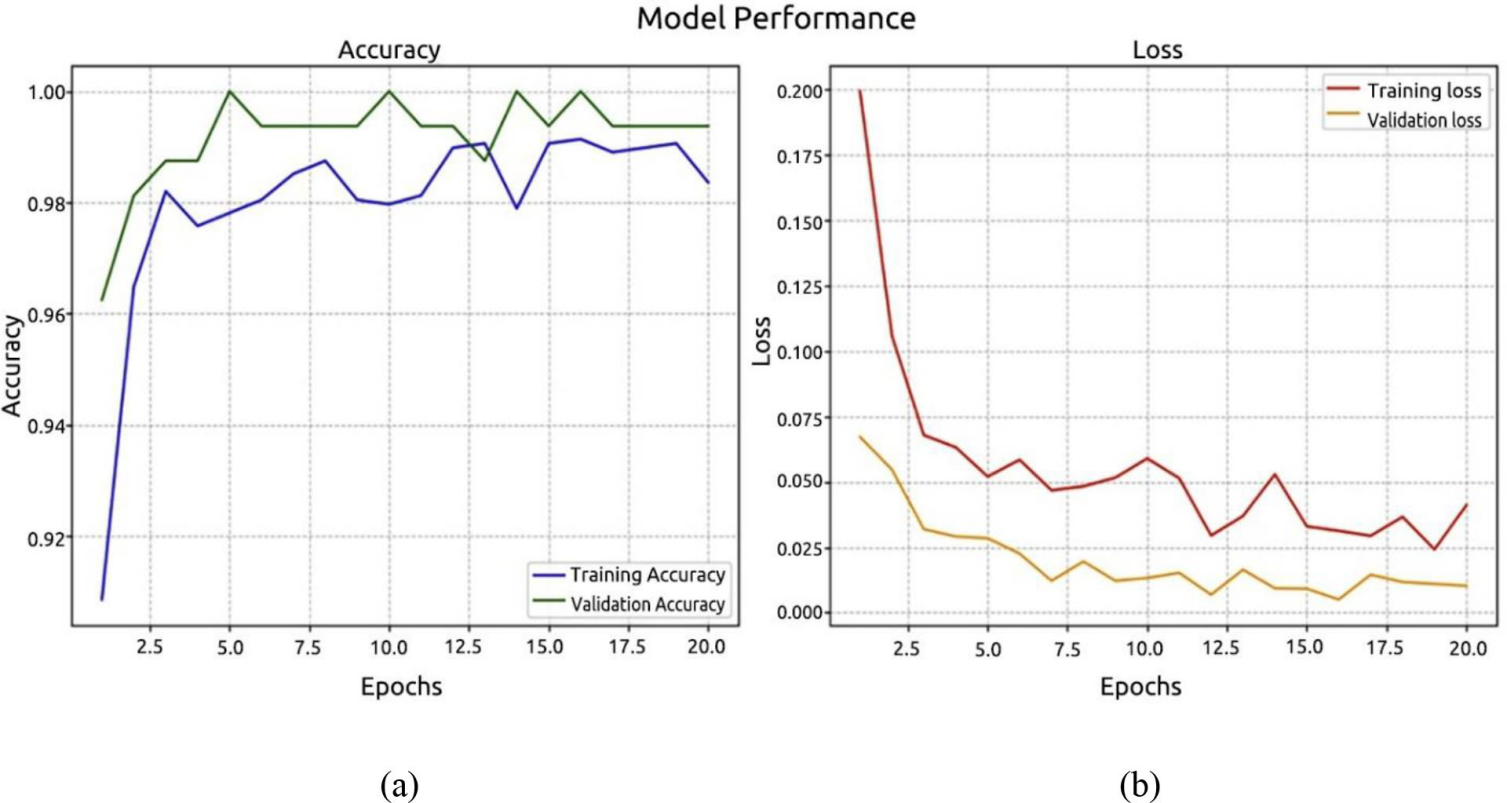
Performance ViT model. **(a)** accuracy; **(b)** loss.

Using the ViT model, the third experiment was carried out. The recognition accuracy graph and the validation and training loss graph are displayed in **Figures 12a, b**, respectively, and they demonstrate the identical methods used to evaluate model loss and recognition accuracy. The model’s accuracy was 99.37%, with a margin of error of 0.01%.

**Figure 13:**
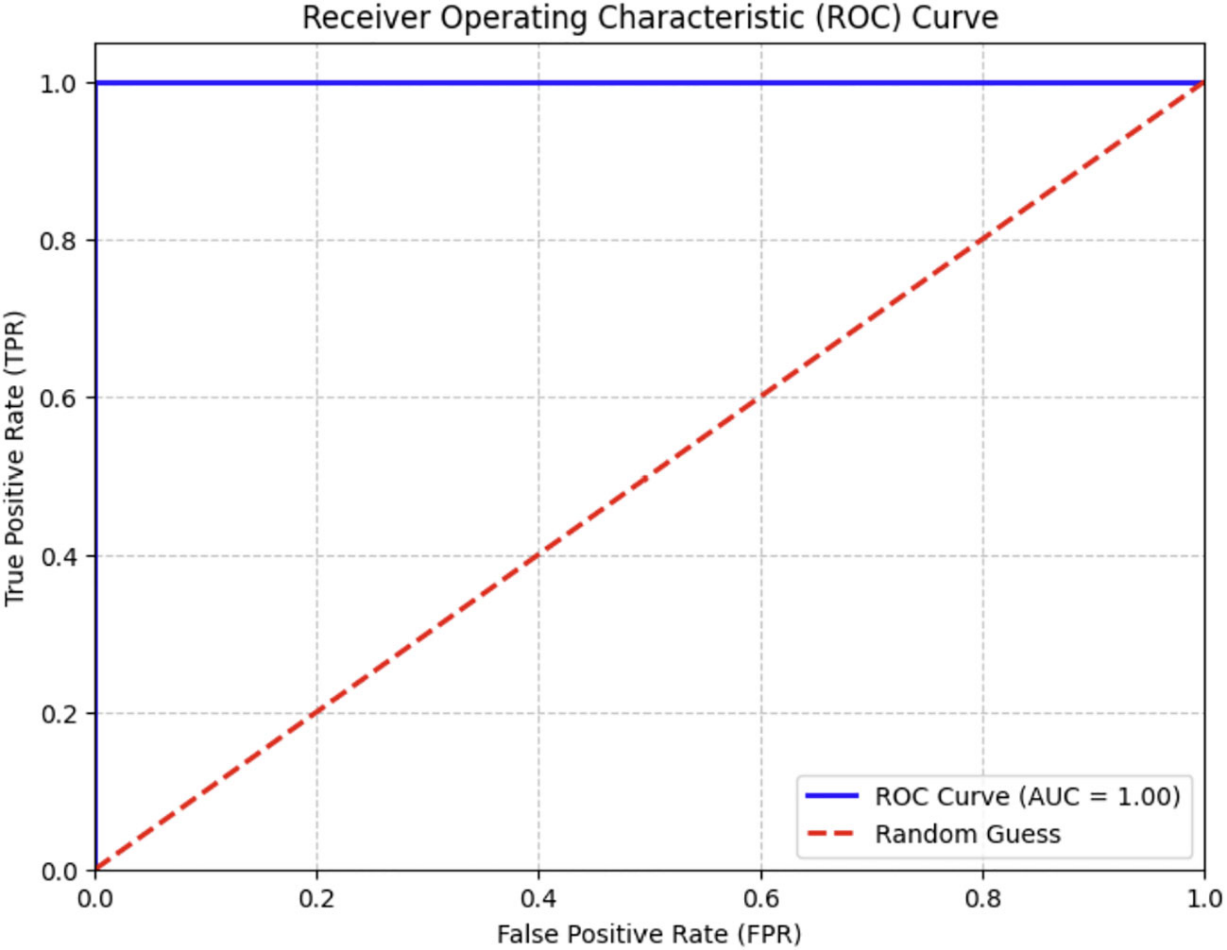
ROC of ViTmodel.

Optimal yields in agricultural production depend on the rapid diagnosis of crop diseases. The early detection of palm diseases using modern technologies is essential for maintaining an enhanced production rate. The literature review indicated that DL models excel in image classification, whereas DL methods effectively reduce training complexity and the need for extensive datasets. We examined three pre-trained models—the EfficientNetV2-B0, the DenseNet-12, and the ViT models—to determine which one was most effective in identifying various palm diseases. The pre-trained models were assessed using assessment criteria, including specificity, sensitivity, and F1 score values. **Table 6** shows the results of the proposed systems against the existing system. We provided a visual representation of the validation accuracy for the pre-trained models by computing the validation accuracy using the F1 score. The ROC curve of the ViT model is shown in **Figure 13**. The model achieved a perfect score.

**Figure 14:**
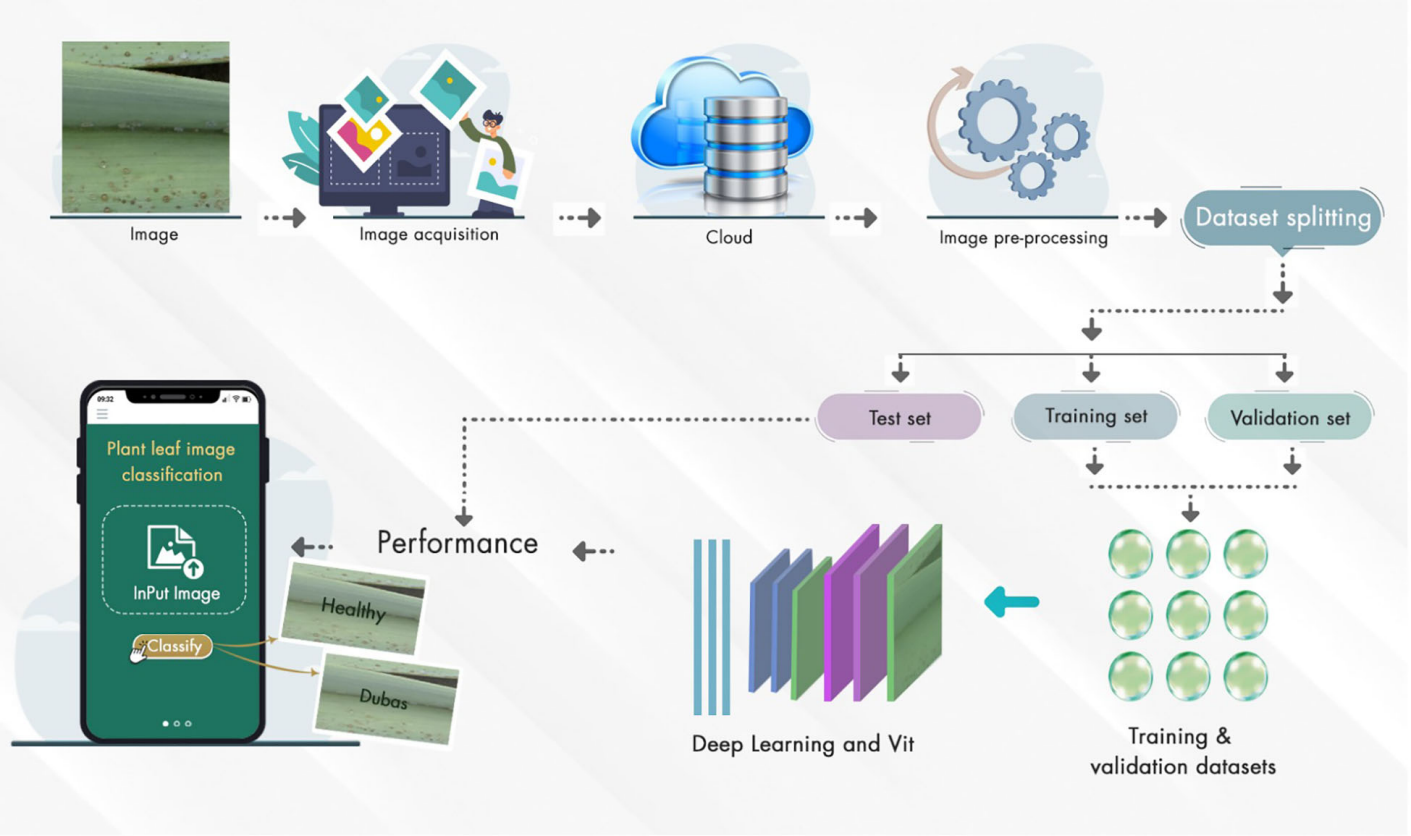
Deployment system for detecting Dubas insect diseases in palm leaves.

Using deep learning and ViT, **Figure 14** shows the method for plant leaf image categorization.

Step 1: Image Acquisition: A digital camera is used to capture plant leaves, both healthy and Unhealthy.
Step 2: Cloud Storage is used to centralize access to the images
Step 3: Pre-processing. Pre-processing of the system is used to handle resizing, normalization, and augmentation, thereby improving model training.
Step 4: Dataset Splitting - The system employs a validation process to divide the dataset into training, validation, and test sets, evaluating the model’s performance.
Step 5: Model Training: The ViT and DL architectures are used to train the model on the training and validation datasets.
Step 6: Performance Evaluation - The trained model is evaluated on the test set, and its classification performance is visualized.
Step 7: Mobile Deployment: Farmers used the mobile applications for classifying plant leaves [*Healthy* vs. *Dubas*-infected] through a user-friendly mobile interface for field use.

**Table 6:** Comparative performance analysis of various network models.

Authors, year	Infested palm	Model	Acc %
Al-Mulla et al., 2023	Dubas	CNN	93-95%
Alshehhi et al., 2022	Dubas	CNN, GoogleNet	98%
Proposed ViT	Dubas	ViT	99.37%

## 5 Conclusion

This study investigated a transfer learning and transformer methodology to provide an edge computing solution for the identification and detection of palm leaf diseases. Python supports three pre-trained models. This study presents a novel method for automatically detecting palm leaf disease against a natural background. This enables the differentiation between groups of infected and healthy leaves. We developed the technique for EfficientNetV2B0, DenseNet12, and the transformer paradigm. The dataset comprises 1600 images of palm leaves, with 800 depicting healthy and 800 depicting Dubas. To reduce computing time, pre-processing was performed, including image resizing and normalization, followed by augmentation. Augmentation was implemented by rotation, flipping, shearing, and zooming methods. The models were used to identify palm leaf disease using the TensorFlow framework with an input dimension of 224 × 224 × 3. The suggested approach achieved superior performance, as indicated by an accuracy value. The experiment demonstrated that the ViT model outperformed the other three models, achieving a validation accuracy of 99.37%, which is comparable to previously published techniques. The developed model successfully sustained elevated recall values, accuracy, and F1 scores. Although several automated detection models for palm leaf disease have been developed, their efficacy has often proven insufficient due to the resemblance of class attributes. This study primarily focused on detecting Dubas insect-related diseases and healthy leaves. This limitation of the study did not include other diseases, such as Brittle Leaves and Brown Leaf Spot.

## Data Availability

The datasets presented in this study can be found in online repositories. The names of the repository/repositories and accession number(s) can be found below: https://www.kaggle.com/datasets/warcoder/palm-leaves-dataset.
